# Understanding staff perspectives of quality in practice in healthcare

**DOI:** 10.1186/s12913-015-0788-1

**Published:** 2015-04-23

**Authors:** Michelle Farr, Peter Cressey

**Affiliations:** Department of Social and Policy Sciences, University of Bath, BA2 7AY Bath, UK

**Keywords:** Customer-oriented bureaucracy, Francis report, Human relations, Patient experience, Quality improvement, Reflection, Services management, Values

## Abstract

**Background:**

Extensive work has been focussed on developing and analysing different performance and quality measures in health services. However less has been published on how practitioners understand and assess performance and the quality of care in routine practice. This paper explores how health service staff understand and assess their own performance and quality of their day to day work. Asking staff how they knew they were doing a good job, it explored the values, motivations and behaviours of staff in relation to healthcare performance. The paper illustrates how staff perceptions of quality and performance are often based on different logics to the dominant notions of performance and quality embedded in current policy.

**Methods:**

Using grounded theory and qualitative, in-depth interviews this research studied how primary care staff understood and assessed their own performance and quality in everyday practice. 21 people were interviewed, comprising of health visitors, occupational therapists, managers, human resources staff and administrators. Analytic themes were developed using open and axial coding.

**Results:**

Diverse aspects of quality and performance in healthcare are rooted in differing organisational logics. Staff values and personal and professional standards are an essential element in understanding how quality is co-produced in everyday service interactions. Tensions can exist between patient centred, relational care and the pressures of efficiency and rationalisation.

**Conclusions:**

Understanding the perspectives of staff in relation to how quality in practice develops helps us to reflect on different mechanisms to manage quality. Quality in everyday practice relies upon staff values, motivations and behaviours and how staff interact with patients, putting both explicit and tacit knowledge into specific action. However organisational systems that manage quality often operate on the basis of rational measurement. These do not always incorporate the intangible, relational and tacit dimensions of care. Management models need to account for these relational and experiential aspects of care quality to support the prioritisation of patients’ needs. Services management, knowledge management and ethics of care literature can provide stronger theoretical building blocks to understand how to manage quality in practice.

**Electronic supplementary material:**

The online version of this article (doi:10.1186/s12913-015-0788-1) contains supplementary material, which is available to authorized users.

## Background

This paper analyses quality from the perspectives of health service staff in a primary care organisation. It illustrates how the professional and personal values and behaviours of staff play a fundamental role in their conception of and co-production of quality in everyday practice. It considers the implications of these findings for the ways in which quality is managed within health systems. Within the field of healthcare quality much work has been focussed on developing and analysing different quality indicators and measures. Less has been published on how practitioners understand and assess the quality of care in routine day to day practice [1]. Indeed it has been acknowledged that:

‘*Robust systems and processes to monitor, manage performance and regulate the quality of care provided to patients are essential. However the success of these is almost entirely dependent on the values and behaviours of staff and organisations working throughout the system’* [2] [p.7].

Managing quality is clearly not all about monitoring systems and regulation, but also concerns health care workers’ values, training and personal behaviours and, importantly, how service quality becomes co-produced in service encounters. We explore these additional elements of ‘quality in practice’ in this paper. This understanding then helps us to consider the different approaches that can be applied to support staff in the development and improvement of service quality. This paper covers three key areas in its background literature. Firstly policies concerning the quality and performance of healthcare are overviewed, analysing these in the broader context of public service reform. Key theoretical influences of these reforms are critiqued, analysing how they have been applied to the management of performance within healthcare. Secondly, alternative conceptions of how quality can be understood and managed from broader literature in services management, knowledge management and ethics of care literature are outlined. Thirdly, studies that theorise and analyse how different elements of quality and performance come together are considered, exploring the dynamics between elements such as person-centred care and efficiency.

### The policy and management of health service quality

Within the NHS in England the accepted definition of quality has three attributes: clinical effectiveness; safety; and the importance of positive patient experiences [2]. The Institute of Medicine provides similar characteristics of quality, including safety, effectiveness, patient-centredness, timeliness, efficiency and equity [3]. Within these definitions there are different aspects of quality where some facets rely on relationally based aspects of care (emerging from staff and patient interactions) whereas others are more functional and transactional and rationally calculable (efficiency, waiting times) [4,5]. Taking a broader perspective the quality agenda in health can be understood in the context of a much wider framework of reforms, within which performance management plays a significant role. Within health policy in England since the 1980s there have been significant shifts toward market based structures that have been accompanied by a considerable set of performance monitoring regimes, embracing new public management (NPM) [6]. In the English NHS the last three decades have seen a substantial shift from allowing professionals autonomy to manage their own performance and quality to the current system today where performance and quality is defined by national policies, and measured through comparative indicators and external standards audited by separate bodies. These reforms were based on the assumption that professionals could not be trusted to manage their own performance, and that the entrenched interests of health professionals jeopardised new quality-related initiatives [7,8].

New public management (NPM) is based upon combining ‘new institutional economics’ (public choice, transactions cost theory and principal agent theory) with managerialism [6], which considers organisations in a detached, rational, scientific way. These theoretical underpinnings of NPM assume that public services can be disaggregated into specific, measurable units and that inputs and outputs (and their costs) can be accounted for and controlled [9], importing management techniques from private sector manufacturing into the public sector [10]. It is assumed that quality of services can be monitored and measured as part of wider performance management regimes that control explicit, quantifiable units. This approach has its basis within a logic of formal rationality [11], based upon the reasonings of rational calculation, linear thinking, and formal measurement as a means of controlling the world. In health this can lead to a focus on quality where that which can be measured is often focussed upon rather than relational, interpersonal and affective dimensions of care [12].

In addition to this overreliance on the measurement of quality, NPM has particular implications in relation to staff motivation. Public choice theory is based on the individualistic assumption that agents are rational, calculating and self-interested [6]. Managerialism is influenced by Taylorist scientific management principles [13]. These aim to increase efficiency and standardisation through the separation of conception and execution of tasks, and the institution of ‘one best way’ determined by scientific procedures [14]. Both public choice and Taylorist principles assume workers have an instrumental, self-interested motivation; they do not account for the fact that people may act morally or ethically. Such theories have been critiqued in relation to the wealth of human psychology evidence [15] that illustrates people are motivated by diverse material, intrinsic, social and normative values [16-18].

Whilst market-based neo-liberal reforms are grounded in these economic assumptions, the extent to which Taylorism is fully applicable to health care practice is debated, given that healthcare staff do retain some autonomy [19]. What has actually happened in the development of healthcare management is that a much broader range of policy and managerial logics have been drawn on, which creates tensions in practice, due to their different underlying assumptions [20]. For example people management practices in health services have been informed by wider human resource management, high commitment management and employee engagement literature [21-25] which contrast with Taylorist principles. There is a growing body of evidence that illustrates how people management practices and employee empowerment and engagement can lead to improved organisational performance and patient outcomes [22,24].

### Alternative conceptions of service quality and its management

In health there is a considerable literature that critiques a NPM market-based approach, commenting on an overreliance on managerial practices based on economics [26] and highlighting the importance of relational care [27] and compassion [28]. These critiques can be augmented with key insights from services management, ethics of care literature and knowledge management. These three different perspectives help us explore the relational aspects of services, where knowledge and resources are practically applied in distinct situations.

New public governance (NPG) advocates the importance of services management literature in enabling a more nuanced understanding of public service processes [9,10,29]. In contrast to NPM’s mechanistic and disaggregated approach, services management literature has advanced a theoretical view of services from a systemic, interconnected approach [30]. Services have been defined as the use of knowledge and skills for another’s benefit. Services management literature highlights how service quality extends from a systemic and relational process that is co-created within the interactions between staff and service users [30]. Expectations and service interactions are central to service quality, and these user experiences at the service interface co-create aspects of service quality [31]. The services management literature conceptualises how value is jointly co-created through collaborative relationships and the application of knowledge rather than through transactions [30].

Turning to a different body of literature, feminist political theories concerning the ethics of care contest the idea that agents are rational, detached and autonomous actors [32], as theorised in neo-liberal public service reforms. In contrast the ethics of care literature conceives of people as connected and interdependent through a ‘relational ontology’ [33]. Influenced by care ethics and feminist political theory, Mol [27] takes an anthropological approach to examine health, care and the body. She examines the care and experiences of diabetes patients in the Netherlands, exploring how social and technical elements of care combine in actual care practices. She delineates what she describes as the ‘logic of choice’ (market based health transactions) from the ‘logic of care’ (an emotional, attuning process) and suggests that ‘good care’ develops from collaborative work between patients and practitioners where knowledge and technologies are adjusted to specific patients’ bio-psycho-social lives [27].

Within health explicit knowledge has been a major priority within evidence-based practice and clinical knowledge. Various separate bodies have developed standards and protocols formalising knowledge at the expense of tacit clinical judgement [34]. However clinical knowledge is embedded and distributed with tacit and experiential elements, and clinicians often use internalised, collective and tacit “mindlines” rather than formally adhering to protocols and standards [35]. In health policy the intangible and intuitive dimensions have received little attention, yet knowledge management practices within the private sector have been increasingly concerned with these less visible elements of knowledge [36]. Managing performance in knowledge work is often based on alignment with the motivation and values of workers, and can include professional networking, the sharing of knowledge, reflective spaces, team development and peer negotiated standards [37]. The use of these approaches within health [38] may begin to address the relational, knowledge in practice elements of interactive health service work, although they may be in contrast to more rational measurable systems that are currently in vogue.

### Managing the different logics in performance and quality

Healthcare performance and quality is multifaceted, and its different aspects can create tensions and contradictions in practice, involving ‘a delicate balancing act’ [39]. These dynamics are modelled in the ‘customer-oriented bureaucracy’ [40] that illustrates how in service organisations the logics of formal rationality [11] and Taylorist mass production encourages efficiency, yet services also require personalisation and an individual orientation. These contradictory dynamics are managed through an appeal to ‘balance’, which creates a ‘fragile order’ [40], as opposed to an acknowledgement of trade-offs between different service logics. Employees who work at the service interface can experience the incongruities of these different forces, as they work with clients. The customer-oriented bureaucracy has been applied to the NHS, highlighting how some clinicians’ desires to meet the needs of patients may be squeezed within health budgets [41]. Each health care intervention needs to be personalised to an individual’s circumstances, and this ‘person-centred care’ is a mark of quality [42]. However nursing staff can face dilemmas, wanting to provide holistic care, but working in a system based on a ‘production line style of care giving’ [43]. The dilemmas of the ‘balance’ between cost-efficiency and patient needs and preferences can be experienced by nurses as a conflict [44]. Organisational constraints can restrain health professionals in their capacity to enact their ideals and professional values, leading in some cases to burnout, disillusionment and intention to leave jobs or the profession completely [45].

The dynamics of the customer-oriented bureaucracy model can be seen through the Francis Inquiry that investigated the failings of care at Mid-Staffordshire NHS Foundation Trust. The Francis Report highlighted how failures of actual patient care were in part due to ‘a focus on reaching national access targets, achieving financial balance and seeking foundation trust status’ [46] [p.3]. Such analysis illustrates how prioritisation of these performance targets and efficiency concerns may be at the detriment of the relational and interpersonal aspects of care that can escape regulatory measures. It has been noted that this focus on key targets was guided by wider policy imperatives at the time [47]. Indeed somewhat prophetically, the Chief Medical Officer Sir Liam Donaldson back in 2005 suggested:

*‘When I express concern about the priority given to the quality of safety of care by NHS managers and boards compared to financial balance and productivity targets, I am told not to worry because performance is judged on a ‘balanced scorecard’. It sometimes feels that the reality is more like a ‘scratch card’ where the money and service activity boxes are revealed but quality and patient safety remained covered over’* [48].

In this study, these dynamics of the customer-oriented bureaucracy were initially considered as significant, and became an important theoretical construct in the analysis as practitioners spoke of their dilemmas in how they managed the different demands of the job.

This research set out to understand how staff relate to different performance and quality regimes, comparing national standards with an analysis of how staff actually enact and interpret performance and quality within their own roles and those of people they managed. The study examined what makes a difference to staff in being able to do a ‘good job’, how they understood and accounted for this, and how they were enabled and motivated to improve the performance and quality of their work*.* The work was informed by the theoretical frameworks and evidence that link people management practices to employee behaviour, organisational effectiveness and patient outcomes [22,24,25]. It adds to the existing literature by exploring how healthcare employees actually enact and interpret performance and aspects of quality within their own roles and those they manage, examining what made a difference to them in being able to do a ‘good job’ and how they understood and accounted for this.

## Methods

This study was based in a Primary Care Trust (PCT), where services were spread over a range of rural and urban areas. The researcher was employed by a University but worked within the Primary Care Trust as a Knowledge Transfer Partnership Associate on a daily basis over a period of two years. The role incorporated the research upon which this paper is based, and also additional human resources and staff development work. The PCT was situated in a geographic area of historical overspend, although the PCT itself had managed to balance its books through a tight financial focus, having made some recruitment restrictions as a result of financial imperatives. Qualitative research explored how both clinical (community and ward based) and non-clinical staff (managerial and administrative) understood and assessed quality and performance in everyday practice, exploring the values and motivations of staff and their relationship to performance and healthcare quality. The research question was:

*How do staff construct, define, understand and assess performance and quality in their roles in everyday practice?*

The project explored professional practices, values and motivations and their relationship to quality and performance measures in healthcare, analysing how understandings of a ‘good job’ develops from the perspectives of staff. A grounded theory approach [49] was adopted, conducting qualitative interviews which focused on people's experiences, perceptions and practice. The research obtained formal ethical approval, REC reference 06/Q2001/34. All participants received written information about the study, and participation was voluntary through written informed consent.

### Sampling

Sampling decisions were agreed at monthly steering meetings. The research focused on job roles where there was greater autonomy in the post (e.g. community based roles) and or where objectives may have been less clear. These criteria were based on the likelihood that there may be diverse conceptions of how job performance may be conceived and completed in these cases. The breakdown of the national staff survey results by occupational group provided additional information to support sampling decisions. For example, health visitors reported working significant extra hours due to the demands of the job; whilst clerical staff had lower results for having clear planned objectives and appraisals. Additional staff who had more stability within their role were also invited to take part in the research to compare findings using negative case analysis [50]. Front line managers of the roles sampled, and more senior managerial roles were also interviewed. A small number of staff groups were chosen to be able to compare findings both within staff groupings and across. 21 people were interviewed by the first author, comprising of health visitors, occupational therapists, managers, human resources staff and administrators. A purposive sample combined variety with opportunities for more intensive study, where there were particular learning opportunities [51]. Community based workers such as health visitors were of particular interest to organisational stakeholders, the additional degree of independence that such workers experienced made their case both theoretically and managerially interesting. Occupational therapists were also prioritised as some staff operated in the community and some on wards, this contrast being used to explore some of the differences between community and site-based work. The sample consisted of front line clinicians (n = 6), non-clinical front line staff (n = 5), non-clinical front line managers (n = 3), clinical managers (n = 3) and senior managers (n = 4). Sample size was informed by ongoing data analysis to clarify and develop insights and tentative theory [52], and stopped when theoretical saturation was reached. It was not possible to follow up those staff who choose not to participate in the research.

### Recruitment and in-depth interviews

To recruit participants the researcher introduced the research aims and objectives to front line and senior managers. The researcher then met with potential groups of staff to explain the aims of the research, and distributed information sheets, inviting potential participants to get involved in the research. Staff responded directly to the researcher to take part, managers did not select participants, and it appeared that staff could talk freely in the interviews as some were critical and outspoken.

An in-depth interview model was followed to understand practitioners’ lived experience, occupational values and perspectives [53]. Interview topic guides were used to steer rather than direct the interview conversations, with space to explore areas of significance to participants, following the path of the interviewee’s dialogue [54]. Interview topic guides were framed through a performance management cycle; covering areas of planning, acting, monitoring and reviewing [55]. The topics included what staff did on a day to day basis, perspectives on the objectives and results that they needed to achieve and how these were set and measured, definitions of a good job, how staff knew they were doing a good job and the evidencing of that quality and service outcomes [see Additional file 1 for example interview questions]. Interviews often expanded beyond these questions and also covered motivation, job satisfaction, reflection, innovation and improvements to services. Interviews ranged in length from 35 to 85 minutes, with an average of around 55 minutes. Interviews were conducted in a private room in the PCT offices. Interviews were audio-recorded with consent, transcribed by the researcher to familiarise herself with the data [49] and anonymised. All interview recordings and anonymised transcripts were stored on the University computing system where only the researcher had access, ensuring confidentiality.

### Data analysis

Following grounded theory techniques the data analysis began as soon as the first interview transcripts were written and further sampling supported the elucidation of ideas and developed provisional theory [56]. Grounded theory was used to analyse the data using an open coding and axial coding system [57]. The first stage of analysis through open coding focussed on scrutinising interview transcripts line by line to identify concepts that fitted the data. Data categories were developed, and axial coding was used to specify the properties and dimensions of particular categories [49]. Front line staff were interviewed first, and some initial themes that emerged from these interviews were then explored with managers through later interviews. The results of the PCT’s national staff survey and organisational documents were also used in the original analysis to complement the interview material, gathering from different data sources to enhance internal validity and provide a degree of triangulation of perspectives and issues arising [52]. In grounded theory existing literature and theory is integrated into research as the data analysis and theoretical categories develop [52]. This iterative approach weaved theoretical categories into the ongoing data analysis with memo writing being used to explore and discover ideas about categories, make comparisons and develop theoretical insights [49]. These theories were then tested out through further sampling and interviews [52]. Before the study began, concepts of motivation, people management practices, performance management and quality improvement were identified as important. As the analysis developed, the theoretical constructs of the customer-oriented bureaucracy [40], knowledge management and services management literature became important theoretical perspectives that emerged. Internal validity was enhanced through the triangulation of perspectives across different staff groups [56]. All participants were contacted at the end of the analysis to present the use of their quotes, which confirmed interpretive validity, with a further conversation with one participant to clarify a particular issue.

## Results

An overview of the detailed findings of this study is now presented, illustrating examples of the different analytic themes that explore how quality in practice develops. Firstly, findings are presented that illustrate the importance of intangible, tacit knowledge in contrast to explicit, standardised knowledge and rational measures. This section explores the relational elements of quality in practice, illustrating how quality is both co-created and perceived in interaction between service providers and patients, yet this phenomenon is not always easily measured. Secondly, the dynamics between efficiency and patient-centred services are explored, analysing how they were experienced in practice by different groups of staff. Thirdly, the importance of collective dialogue and reflective spaces are analysed as an important arena for staff to be able to discuss and develop practice to improve quality. Together the findings illustrate the importance of a diversity of mechanisms to manage quality in healthcare practice, ensuring a greater focus on the relational and intangible elements of quality as well as current quantitative measures.

### How staff understand quality in practice

Whilst health systems tend to focus on measurement as a driver and arbiter of dimensions of quality, when asking staff how they understood performance they spoke of the tacit and experiential aspects of the clinical decision making and quality of care:

*‘I use my own experience and my clinical reasoning to think that actually that person isn’t well enough to be seen yet, and that that person is right. I can’t …it is very hard to say exactly… Oh I used this, that and the other today, you can’t, because each person is an individual and a lot of it is experience.’* (Interviewee 07, front line clinician).

In addition to the importance of these tacit clinical understandings, values and normative standards also played a role in understanding how and when to intervene to ensure appropriate outcomes. For example within health visiting, professionals need to appropriately assess parenting skills:

*‘I constantly find a problem between ‘is parenting good enough?’…When have they fallen below that? How does one define that?… I have to look at my own values and decide, ok it’s not what I would do but it isn’t doing any damage to the child and they love that child.’* (Interviewee 10, front line clinician).

Indeed individual staff may have different standards, partially based on clinical training and professional roles, but also extending to personally based standards. This element of personal standards was apparent across all job roles, whether clinical, managerial or administrative:

*‘I suppose it all boils down to personal standards, there’s a standard that I can’t justify, I can’t say this is why I’ve set my standard this high, or this low or whatever but there’s a standard and I like to work towards that standard. I think we all have different standards at different things… You ask me to try and rationalise it, I can’t, it’s just my, it’s just me, it’s just my standard.’* (Interviewee 12, non-clinical front line).

*‘I think quality indicators are quite an individual thing actually for clinicians, I think different people depending on where they’ve come from and probably different professions, would have a different notion of what a quality indicator would be.’* (Interviewee 18, clinical manager).

Not only were personal staff standards important but so were those of patients. It was illustrated how quality measures and professional standards may not always align with individualised, patient-centred approaches that are led by patients’ values and concerns. This was brought to life in an example that one interviewee gave where aspects of person-centred care clashed with particular professional practices. Here, when nursing a sick child with a terminal illness, they spoke of how their care was sensitively discussed with the family. Here aspects of quality were co-created in discussions between professionals and the patient and family. The wishes of this patient and family were not always aligned with particular nursing practices, for example not always wanting to be tidy with brushed hair. When a different clinician intervened she differed with the patient-centred approach negotiated with the family, following her own, different professional standards. Here both standards may be appropriate at different times. This example illustrates in practice how quality can be co-produced through dialogue at the service interface, and that quality extends from the interaction and conversations between practitioners and those who are using a service. Overall the findings illustrate the importance of staff values, attitudes and standards in co-producing quality in everyday service interactions, alongside ongoing communications with the users of a service.

### Tensions between patient-centred care and efficiency pressures

The study was set within a wider context where there were severe financial pressures on the Trust.

*‘There is no slack in the system and actually trying to maintain clinical quality has been really, really hard. I think the actual driver from the government is maintaining financial balance’* (Interviewee 20, Senior manager).

*‘You are making every pound count, and stretching it as far as you possibly can. And it comes down to some really difficult decisions that are having to be made, not only when you are a front line health care professional, right through to as an organisation, do we continue to commission x service. So it spans across, no matter what you do.’* (Interviewee 15, Senior manager).

Within this context the pressures of diverse aspects of quality played out in practitioners’ everyday practices. Both managers and practitioners spoke of the tensions apparent between aspects of quality and efficiency:

*‘People are still desperately trying to deliver a quality job, all the pressure is on efficiency.’* (Interviewee 18, clinical manager).

For example, clinicians spoke of the dilemmas they faced in working with large numbers of patients, whilst ensuring sufficient time was spent with each to provide the most effective service. Where there were financial constraints this could strain staff’s capacities to deliver the levels of quality of care that they valued as part of their professional expertise.

*‘Staff don’t always feel able to deliver the service that they think they should be providing and that is because we are living within the financial framework that we live within.’* (Interviewee 15, senior manager).

However some managers and clinical staff felt that due to resource pressures, this was the time to really reflect and use the situation to create necessary changes and improvements. For example one clinician discussed ways to prevent hospital admission through earlier crisis intervention:

*‘Some of the best ideas come out of financial restraints. And this is why I am saying that we should be thinking of different ways of doing things.’* (Interviewee 10, front line clinician).

Not only were there increased financial pressures, but staff also spoke of increasing patient expectations:

*‘They’re not necessarily the grateful patient that we perhaps used to have.’* (Interviewee 18, clinical manager).

When staff did find it difficult to ‘balance’ the different logics of efficiency and user centred needs, job satisfaction could diminish:

*‘I do get very dissatisfied if I can’t do, give the support that I would like to… because of the time pressures really.’* (Interviewee 11, Front line clinician).

*‘The priorities have changed; I keep going back to that it has changed. If I really sat and thought about it I would probably get quite miserable that I don’t give as a good a service as I previously gave to lots of people.’* (Interviewee 12, Non-clinical front line staff)

Staff exhibited a great degree of intrinsic motivation, wishing to embody their professional and personal values within their work and ‘*make a difference*’ (Interviewee 13, Non clinical front line). People’s sense of job satisfaction was highly connected to their motivations for going into their professions.

*‘I think the biggest thing is helping others’* (Interviewee 04, Non clinical front line).

*‘You are motivated because you are helping people, you are getting them back to a certain level of independence’* (Interviewee 09, Front line clinician)

When staff were then working within a context where prioritisation and time management were key, where *‘complete total care’* (Interviewee 18, clinical manager) may have been difficult within the restricted resources, this could affect the pursuit of the embodiment of professional and personal values.

*‘What will happen is with clinicians is they get to a point where the manager says no just leave them but actually then they’ll get into feeling that that means I’m not doing a good job and I feel I can’t perform my professional role and so then there’s you can’t leave it go, you can’t leave x, y or z not done…. However much you’re saying prioritise and leave things go, people are just going to feel disillusioned and dissatisfied because that’s not what they’ve come into this work to do… So they’ll keep on doing it to a point until they just get burnt out’* (Interviewee 18, clinical manager).

### Enablers to understand and improve quality in practice

Staff spoke about the importance of reflective space and peer discussions in helping to understand and reflect upon their working practices with patients. Autonomous practitioners who worked in communities shared cases as a means to gain peer feedback:

*‘We share what we are doing with clients so that we can work interchangeably and that is very helpful to us, getting feedback from each other, getting responses from the clients.’* (Interviewee 10, front line clinician).

Reflective practice was seen as a way that staff could assess their own performance and quality within their own practice, using both evidence and reflection. Another spoke of how reflective discussions could also support efficiency, highlighting how in an action learning set a conversation enabled staff to ‘*drill down*’ to more effective ways of working together. Processes of reflection within the organisation varied according to professions; some had embedded models and specific reflective spaces, although on occasion these had stopped due to resource pressures, and there were some informal processes at a peer level.

*‘Some of it I think is definitely cultural so there are individuals and professions who have been more used to doing that sort of thing [reflective practice] in terms of saying yes we feel this is a priority for some of our time and other teams where it hasn’t been the cultural norm and they are so busy doing their day to day they can’t possibly stop for half an hour, do reflective practice, because that’s half an hour when they are not doing something else.’* (Interviewee 17, Senior manager).

Ward-based staff found it problematic to carve out a space for meeting as some staff always needed to be on the wards. The organisation had also recently introduced reflective practice sessions for managers, one manager noting that such spaces were more *‘legitimate’* in clinical work yet they were equally relevant for managerial practice.

*‘I think sometimes you lose sight of the fact that we equally need to have that time to reflect as well’* (Interviewee 15, non-clinical manager).

Other interviewees spoke of the difficulties in creating time and space for such conversations within the difficult financial climate and the need for such conversations to be focussed on solutions.

## Discussion

The overview of the detailed findings of this study has analysed how different healthcare staff construct, understand and assess performance and quality in everyday practice. This discussion follows the structure of the data analysis sections; firstly focussing on staff understandings of quality in practice, secondly exploring the tensions that exist between different aspects of performance and quality, before considering how staff may be enabled to improve quality. Firstly this paper illuminates the importance of the tacit, intangible and relational dimensions of quality in actual practice. Staff values and personal and professional standards are core to understanding how quality is co-produced in service interactions. Professional experience, tacit clinical knowledge, personal standards and values, and conversations with patients and families all contributed to how staff understood and assessed the quality of their work in everyday practice. These interactions mirror the conceptualisation of the service process within services management literature and the new public governance paradigm as opposed to the transactional, rational approach favoured in NPM. The findings illustrate that the mechanistic, target, measurement culture that exists needs to be augmented with a far greater emphasis on the social, emotional and relational aspects of care giving and receiving. Whilst Gabbay and May [35] highlight the use of ‘clinical mindlines’ in clinical decision making, that are based on internalised collective and tacit aspects of knowledge, this study extends this notion of the tacit and collective in the accounting for and understanding of performance and quality within health services.

Secondly the data analysis demonstrates how there are tensions between different aspects of quality and performance in health systems, where patient centred, relational ‘*complete total’* care and the pressures of efficiency and rationalisation can sometimes operate as divergent logics within an organisation. These findings mirror the pressures theorised in Korczynski’s customer-oriented bureaucracy model (COB) [40] and extend its application. Whilst Korczynski’s model focuses on the effects of these tensions on front line service workers, the interviews in this study illustrate how these tensions are felt throughout an organisation, including in non-clinical staff, front line and senior managerial positions. However the COB model [40] does not answer the important question of how these inherent tensions between efficiency and customisation can be negotiated in a way that is conducive to the economic needs of productivity, workers’ needs for meaningful and productive work that embodies professional values and service users’ and citizens’ needs for responsive, effective and high quality health services. This study illustrates how staff need collective space to discuss the dynamics of these different tensions and logics in their work. Whilst Evans highlights how strong professional structures and processes are important to help clinical staff manage the inherent anxieties and tensions in their work and to support compassionate care [58], this study illustrates that such support may be needed throughout organisations in both clinical and non-clinical roles. Good staff management, where staff feel valued, supported and engaged is connected to quality of care [59]. The Berwick report [60] emphasised the need to engage and value staff, fostering their growth and development, offering more support and an open culture to improvement. This paper extends this by suggesting that health service work has considerable similarities to work in knowledge intensive and service industries and that practices from knowledge management, services management and ethics of care literature can support Berwick’s goals. The data findings in this study show that staff value reflective processes where they can have space to discuss quality in practice and that extending such processes to managerial roles may also be of benefit. Whilst such spaces may get squeezed by the pressures of service delivery and resource constraint, there are several reflective models that have been developed and implemented within different parts of the health service such as communities of practice [38,61,62]. These collective reflective processes can support the development of work practices and support personal engagement in the workplace [63], Bate and Robert [36] suggesting that developing ‘quality communities of practice’ can facilitate collective learning and improvement within the health service.

In the Francis report there are substantial recommendations that the recruitment, education, and training of clinical staff be enhanced with discussion of ‘*appropriate values, attitudes and behaviours*’ [46]. However there is less emphasis in the Francis report on how these values, motivations and commitments of staff can be supported in day to day practice, when the stresses of service delivery and complex, diverse pressures are apparent. Indeed it has been suggested that the importance of contextual factors have not been given sufficient attention in the wake of the Francis report to enable an understanding of how complex contexts might affect actual compassionate behaviour in day to day practice [64]. Whilst the Francis report recommends that cultural measurement tools such as a *‘cultural barometer’* might be developed to monitor the *‘cultural health’* of health service organisations, it has been commented that such solutions of more measurement may not necessarily produce the outcomes intended, as *‘culture itself becomes a target driven priority’* [65] [p.3]. This study demonstrates how the management of quality in healthcare needs to extend beyond formalised policies and measures to acknowledge how care is a context-dependent and relational process. It argues against more measurement and suggests that where aspects of quality are less easy to measure, management mechanisms based on trust and values may be appropriate [66]. It supports Ballatt and Campling’s [67] notion of the importance of the values of interdependency and connectedness in the organisation and management of the NHS. This paper illustrates that there is a strong body of theoretical literature encompassing services management, knowledge management and feminist perspectives of care that can inform this development.

The limitations of this study are that it focussed on a small sub-set of health professionals due to resource constraints. Whilst a level of theoretical saturation was achieved in the staff sub-groups chosen, further work would be beneficial to comparatively understand how a wider range of different clinical professions understand and assess the quality of their service provision in day to day practice in different health service organisations. However despite this in-depth qualitative research being done on a small scale, the findings align with other work in this area that illustrates how reconciling the different pressures of the formal rationality of cost-efficiency and ‘relational’ holistic patient care can cause dilemmas in practice [41,43,44,68,69]. In addition, it resonates with and extends the work of Gabbay and le May [35] highlighting the importance of the collective and tacit aspects of knowledge, not only in clinical decision making, but in the understanding and accounting for quality and performance in healthcare.

## Conclusions

There are divergent logics and tensions within different aspects of quality, where patient-centredness requires specific attention to individual needs, and efficiency which can be supported by rationalisation and mass production. These different logics can be experienced as dilemmas in clinicians’ everyday practices. Whilst quality systems often operate according to that which is measureable, the difficulty of this approach is that the intangible, relational and tacit elements of care become less visible within health systems. It has been exemplified through this paper that services and knowledge management practices and ethics of care literature have important contributions to make to the quality agenda within health services. Ethics of care theory [32,33] begins from a relational, interconnected ontology as opposed to that of an autonomous rational actor. This relational ontology can provide a stronger grounding logic for developing organisations on a basis of interpersonal care. Services management literature highlights the importance of how quality is co-produced in everyday service interactions between staff and service users through a relational process. Knowledge management literature provides a range of tools and techniques that can be used to manage and support quality, including reflective spaces, communities of practice and collective learning. It has been illustrated that these approaches may align with elements of staff values, motivation and commitment to professional learning and development, complimenting rationalised measurement systems to support the intangible and tacit dimensions of quality. Theories that build on understandings of care as a connected, interdependent and relational process [32,33] may provide stronger building blocks for achieving that which Robert Francis identifies as essential within the health service; to improve care and put the patient first [46].

## Additional file

Additional file 1:
**Example interview questions – Front line Manager.**

## Abbreviations

COB: Customer-Oriented Bureaucracy
NHS: National Health Service
NPG: New Public Governance
NPM: New Public Management
