# Supplementary material for: Understanding staff perspectives of quality in practice in healthcare
Source: BMC Health Serv Res. 2015 Apr 23;15:123. doi: 10.1186/s12913-015-0788-1 (PMC4407418; doi:10.1186/s12913-015-0788-1)
Supplement: Additional file 1: — Example interview questions – Front line Manager. [file 12913_2015_788_MOESM1_ESM.pdf]

Understanding staff perspectives of quality in practice in healthcare

Michelle Farr and Peter Cressey

Additional file 1: Example interview questions - Front Line Manager

1. **Defining the job of frontline staff**
    a. What are the main objectives of the job?
    b. What are the main priorities?
    c. What does the jobholder do on a day to day basis?
    d. What are the main results that need to be achieved through the job?
    e. How are these results managed and/or measured?

2. **How the job becomes defined**
    a. How does the job holder know what to do in their job? How does the job get defined?
    b. How are objectives and targets set?

3. **Job Description and Allocation of Time**
    a. Looking through the job description how would you expect a post holder to be spending their time on the various parts of their job? What are the main priorities?

4. **What's a good job?**
    a. What is a good job in the context of this role? What's your vision of a job well done?
    b. Do you think is an achievable vision in the current context?

5. **Alignment through the organisation**
    a. How do you see the post and the work of your wider team connected to the wider strategic objectives of the PCT?

6. **How do staff know they're doing a good job?**
    a. In this job how would a member of staff know they're doing a good job?
    b. What would be the key indicators of success?
    c. Are there quantitative/ qualitative criteria that could be used to assess how they are doing a good job?
    d. How do you know the role holder is doing a good job?
    e. What sort of performance information can/ do you supply to commissioners?
    f. One of the key areas that staff see as performance indicators is client outcomes and positive change in the patient. However most have found it difficult to provide evidence for this. Do you have any outcome measures? Would something like a health related quality of life questionnaire be useful?

7. **Quality and efficiency agendas**

In the interviews that I have analysed with clinical staff one of the major issues that has arisen is the different demands between clinical effectiveness and quality and productivity/ efficiency especially in the light of the recruitment restrictions.
    a. What do you see as the major issues with respect to this?
    b. How do you see these being balanced in the everyday work of staff?
    c. What would support staff in managing these conflicting demands?

8. **Your job as a front line manager**
    a. How do you balance the needs of your front line staff with the needs of senior managers?
    b. What motivates you in your role? What aspects of your role give you most job satisfaction?
    c. How do you know you're doing a good job?
